# Urinary tract infection due to *Fusarium oxysporum* in an immunocompetent patient with chronic kidney disease

**DOI:** 10.7555/JBR.32.20160128

**Published:** 2017-06-10

**Authors:** Seema Khetan, Prakash Khetan, Venkatesh Katkar, Minal Kusulkar

**Affiliations:** Department of Microbiology, Government Medical College, Hanuman Nagar, Nagpur 440009, India.; Department of Microbiology, Government Medical College, Hanuman Nagar, Nagpur 440009, India.; Department of Microbiology, Government Medical College, Hanuman Nagar, Nagpur 440009, India.; Department of Microbiology, Government Medical College, Hanuman Nagar, Nagpur 440009, India.

**Keywords:** urinary tract, fusariosis, *F. oxysporum*

## Abstract

Infections due to *Fusarium* species are collectively referred to as fusariosis. *Fusarium oxysporum* has been reported to cause keratitis, onychomycosis, skin infections, catheter associated fungemia and has not been described as a cause of urinary tract infection. Here, we present the first case of fusariosis with urinary tract involvement in a 67 year old male, with chronic kidney disease and type 2 diabetes mellitus. This case illustrates the ever increasing spectrum of rare but offending pathogenic fungi. Early diagnosis of infection with a specific pathogen may lead to changes in antifungal therapy and may be critical for an improved outcome.

## Introduction

Fusarium species is a well-known plant pathogen, seen in an immunocompetent patient with soil and water worldwide^[1]^. *Fusarium* species cause a broad spectrum of infections in humans, including superficial infections, as well as locally invasive and disseminated infections^[2]^. It causes invasive infections in immunocompromised patients, especially in bone marrow transplantation and long-term steroid therapy patients^[1]^. There has been a marked increase in opportunistic infection by fungal pathogens involving the urinary tract over the past decade^[3]^. Fungal urinary tract infections are most commonly caused by *Candida* species followed by *Cryptococcus*, *Coccidioides*, *Aspergillus*, *Histoplasma* and *Curvularia* species^[3]^. However, *Fusarium* species has rarely been previously reported as pathogenic fungi in the urinary tract in the English literature^[4]^.

## Case report

A 77 year old male, with type 2 diabetes mellitus for 25 years, complained of dysuria, weakness and decreased appetite for 6 days and fever for one day.

He was diagnosed with chronic kidney disease 15 years ago and received conservative management. There was no history of burning micturition or abdominal pain or haematuria. On examination, the patient was alert, and febrile; his pulse was 100 per minute, and his blood pressure 140/90 mmHg. Systemic examination did not reveal any abnormality.

### Laboratory investigations

**Fig.1:**
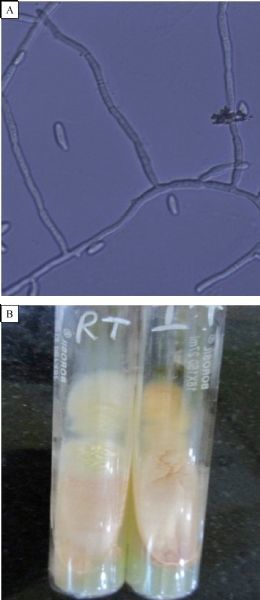
Urine microscopy & culture. A: Urine routine microscopy showing hyaline septate hyphae and fusiform macroconidia; B: SDA showing velvety pale yellow to peach colonies on obverse and pale yellow on reverse. High power magnification 40×.

**Fig.2:**
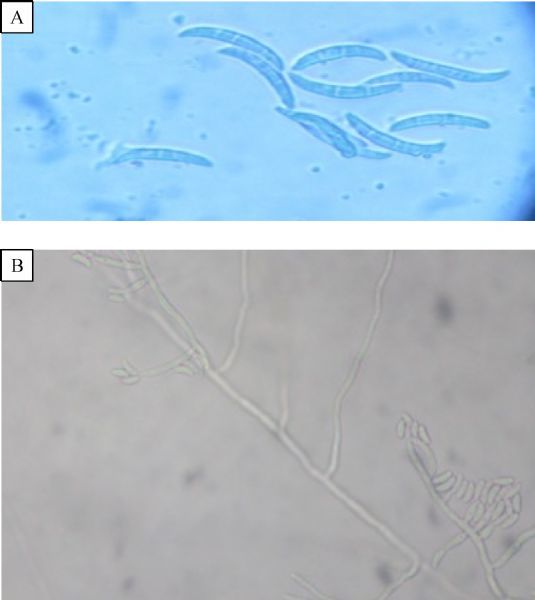
Microscopic pictures of fungal growth. A: LPCB mount showing septate hyphae and sickle shaped macroconidia (3-8 ×11-17 μm) with 3–4 septae under 40× magnification. B: Septate branched hyphae with lateral monophialide conidiophore under 40× power.

**Fig.3:**
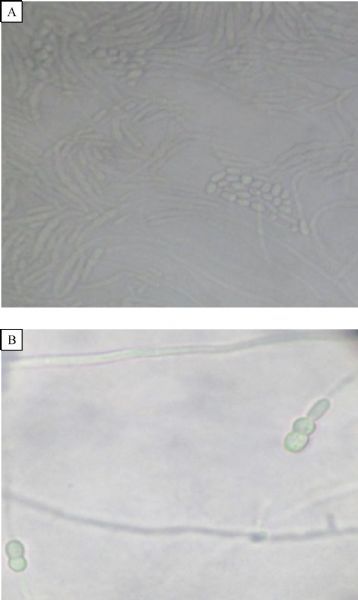
SDA slide culture. A: Microconidia mostly non-septate, oval to ellipsoidal on SDA slide culture; B: Chlamydospores are terminal, hyaline, smooth or rough-walled, 5–13 μm seen on slide culture.

His laboratory findings included hemoglobin 8.0 mg/dL (normal range 14.5–16.5 gms/dL), total leucocyte count 8,000/mm^3^(normal references 4,000–11,000/mm^3^) with differential count of polymorphonuclear cells 70%, lymphocytes 24%, eosinophils 3%, and monocytes 3%. His blood tests for HIV/HBsAg were negative, fasting blood sugar was 230 mg/dL (normal references 60–110 mg/dL), urea 59 mg/dL (normal references 20–40 mg/dL) and creatinine 2.1 mg/dL (normal references 0.6–1.2 mg/dL). Urine microscopic examination showed occasional leucocytes, septate hyphae and many sickled shaped to fusiform macroconidia with 3–5 septa (***Fig. 1A***). RBCs or organisms were not present. Periodic acid Schiff (PAS) stain was performed on deposit from centrifuged urine sample and showed PAS positive multiple sickled shaped fungal elements in each field. A provisional diagnosis of fungal infection was done due to presence of septate fungal hyphae and fusiform macroconidia in urine wet mount preparation on two occasions. On the basis of urine examination, Fusariosis of the urinary tract was suspected and informed to the clinician. Urine was cultured using standard techniques for the isolation of aerobic bacteria on Blood agar and MacConkey agar. Fungal culture was done on Sabouraud dextrose agar (SDA) supplemented with gentamicin and chloramphenicol. Two slants of SDA for fungal cultures were incubated at 25 ˆC and 37 ˆC. Blood culture was performed to rule out bacteremia and fungemia. Urine culture was negative for bacteria but was positive for fungus after 3–4 days of incubation. On SDA, the isolate produced floccose colonies with sparse to abundant aerial mycelia, initially white, becoming peach/pale orange with both at 25 ˆC and 37 ˆC (***Fig. 1B***). The reverse of the colony was pale yellow to orange with no pigment. On lactophenol cotton blue preparation of growth, septate hyphae and sickle/banana shaped macroconidia with 3–4 septae were seen (***Fig. 2A***). For confirmation of fungal identification, slide culture was done, which showed septate branched hyphae that produced microcondia and macroconidia. Conidiophores were short with single lateral monophialide (***Fig. 2B***). Macroconidia were fusiform, slightly curved to straight, pointed and mostly had three septa. Microconidia were abundant but never in chains, mostly non-septate, oval to ellipsoidal, straight or often curve (***Fig. 3A***). Chlamydospores were terminal, hyaline, smooth or rough-walled (***Fig. 3B***). Thus, the isolate was identified as *Fusarium oxysporum* on the basis of characteristic-floccose pale orange colored colonies, short, single conidiophore with lateral monophialides, fusiform, slightly curved to almost straight macroconidia, oval-ellipsoid non-septate microconidia, terminal oval chlamydospores.^[4]^ Fusariosis of the urinary tract due to *Fusarium oxysporum* was confirmed. Blood culture was negative for bacteria as well as for fungus.

After inception of microbiology report of fungal growth, antifungal treatment was started. On the basis of efficacy and safety considerations, the patient was started on voriconazole 200 mg orally every 24 hours.

Clinical improvement occurred after initiation of antifungal treatment, and thus was continued for 4 weeks. On follow-up after 4 weeks, urine microscopy was normal.

## Discussion

Infections due to *Fusarium* species are collectively referred to as fusariosis^[3]^. The clinical manifestations of fusariosis range from a localized infection (e.g. keratitis, onychomycosis) to disseminated infection involving skin, bloodstream, lung, eye (e.g. endophthalmitis), bone and brain^[5]^. *Fusarium* species are very common in the tropical and subtropical areas^[6]^. Most cases of human fusariosis are caused by *F. solani, F. oxysporum,* and *F. moniliforme* but various other atypical strains are emerging as an important cause of infection, especially in patients having one or the other underlying risk factors like immunosuppression, tissue damage and neutropenia^[2]^. Recently, a case of urinary tract infection due to *Fusarium proliferatum* was published in a patient of agranulocytosis^[7]^. The diagnosis of *Fusarium* infection requires cultures for accurate identification. On slide culture, *Fusarium* species appears as septate hyaline hyphae with acute angle branching. The appearance of banana or sickle-shaped macroconidia on lactophenol preparation is a pathognomonic feature of *Fusarium* species.

In the literature, *Fusarium oxysporum* has been reported to cause keratitis, onychomycosis, skin infection, and catheter associated fungemia^[8]^. *F. oxysporum* has been isolated from hospital water system also^[9]^. Su *et al.* (2007) diagnosed fusariosis in urine in an adult patient with nephrolithiasis but did not mention species. Perinephric abscess in an immunocompetent child has been described by Sidhu S *et al.* (2013) and who identified *F. Chlamydosporum* as the causative agent.

We report a rare case of urinary tract infection due to *Fusarium oxysporum* in a chronic kidney disease, diabetic patient who was also diagnosed with multiple myeloma. To the best of our knowledge, this is the first case described in the literature in which localized urinary bladder fusariosis was present as a cause of urinary tract infection. *Fusarium* species are relatively resistant to treatment with antifungal agents. Amphotericin B is the drug of choice but high doses are needed, which, however, may increase ocurrence of side effects. Voriconazole was reported as a successful treatment of disseminated fusariosis in patients with hemato- oncologic malignancies or refractory fungal infections^[10]^.

Fusariosis of the urinary tract in this patient might have resulted from ascending infection due to unknown source, probably contaminated water source or urinary catheterization. Patient responded to treatment with voriconazole with an excellent outcome and without adverse events. Demonstration of typical septate hyphae or growth of *Fusarium* from clinical specimens remains the gold standard for a definitive diagnosis; hence, PCR analysis of the isolate was not done.

In conclusion, urinary tract fungal infections in immunocompromised patients caused by molds other than Aspergillus have increased. *Fusarium oxysporum* may be an emerging pathogen in urinary tract infection, particularly in immunocompromised patients. Rapid and reliable diagnostic methods for mold identification from various specimens would increase diagnostic precision and should be adopted in routine microbiology laboratory. Judicious use of voriconazole becomes a very important tool in the treatment of this type of infections because of the safety and efficacy of the drug.
